# Supplementary material for: Deletion of wfs1 Impairs Oligodendrocyte Precursor Cells Dorsal Distribution and Myelination Through the wfs1-hmgcs1 Axis in Zebrafish
Source: Biology (Basel). 2026 Aug 16;15(16):1402. doi: 10.3390/biology15161402 (PMC13510151; doi:10.3390/biology15161402)
Supplement: Supplementary file 1 [file biology-15-01402-s001.zip › File S1-Transcriptome sequencing data and differentially expressed genes (DEGs) in wfs1-- zebrafish at 5 dpf/1.GO/wfs1KOvsWT/GSEA/WT/NEUROMUSCULAR_PROCESS/NEUROMUSCULAR_PROCESS(GO_0050905).html]

Details for gene set NEUROMUSCULAR\_PROCESS(GO:0050905)[GSEA]


|  || Dataset | deg.sample |
| Phenotype | sample.cls |
| Upregulated in class | WT |
| GeneSet | NEUROMUSCULAR\_PROCESS(GO:0050905) |
| Enrichment Score (ES) | -0.5626473 |
| Normalized Enrichment Score (NES) | -1.7323923 |
| Nominal p-value | 0.0 |
| FDR q-value | 0.34965762 |
| FWER p-Value | 0.811 |
Table: GSEA Results Summary

  

Fig 1: Enrichment plot: NEUROMUSCULAR\_PROCESS(GO:0050905)
  
Profile of the Running ES Score & Positions of GeneSet Members on the Rank Ordered List

  

| PROBE | DESCRIPTION   (from dataset) | GENE SYMBOL | GENE\_TITLE | RANK IN GENE LIST | RANK METRIC SCORE | RUNNING ES | CORE ENRICHMENT || 1 | 83413 | glra4a |  |  | 765 | 2.446 | 0.1162 | No |
| 2 | 563467 | slc17a8 |  |  | 5783 | 0.608 | -0.0064 | No |
| 3 | 557263 | trrap |  |  | 7981 | 0.425 | -0.0510 | No |
| 4 | 103911405 | otog |  |  | 10770 | 0.257 | -0.1238 | No |
| 5 | 436944 | clrn1 |  |  | 11877 | 0.197 | -0.1472 | No |
| 6 | 564412 | ush1c |  |  | 13432 | 0.119 | -0.1891 | No |
| 7 | 553460 | snap29 |  |  | 14882 | 0.052 | -0.2316 | No |
| 8 | 100331283 | otofb |  |  | 17433 | -0.051 | -0.3087 | No |
| 9 | 100330314 | ush1ga |  |  | 20427 | -0.184 | -0.3921 | No |
| 10 | 407978 | cdh23 |  |  | 22950 | -0.323 | -0.4527 | No |
| 11 | 554164 | agtpbp1 |  |  | 24604 | -0.422 | -0.4804 | No |
| 12 | 403310 | cacna1da |  |  | 24910 | -0.442 | -0.4647 | No |
| 13 | 557476 | otofa |  |  | 25198 | -0.459 | -0.4473 | No |
| 14 | 335756 | wrb |  |  | 27156 | -0.643 | -0.4719 | No |
| 15 | 114457 | atp1b2b |  |  | 30050 | -1.265 | -0.4902 | Yes |
| 16 | 100002872 | cyfip2 |  |  | 30090 | -1.280 | -0.4180 | Yes |
| 17 | 368439 | mycbp2 |  |  | 30473 | -1.490 | -0.3446 | Yes |
| 18 | 544664 | erbb3b |  |  | 31538 | -3.021 | -0.2049 | Yes |
| 19 | 541552 | eya4 |  |  | 31710 | -3.769 | 0.0056 | Yes |
Table: GSEA details
[plain text format]

  

Fig 2: NEUROMUSCULAR\_PROCESS(GO:0050905)
  
Blue-Pink O' Gram in the Space of the Analyzed GeneSet

  

Fig 3: NEUROMUSCULAR\_PROCESS(GO:0050905): Random ES distribution
  
Gene set null distribution of ES for
 **NEUROMUSCULAR\_PROCESS(GO:0050905)**
